# Neuronal signals regulate obesity induced β-cell proliferation by FoxM1 dependent mechanism

**DOI:** 10.1038/s41467-017-01869-7

**Published:** 2017-12-05

**Authors:** Junpei Yamamoto, Junta Imai, Tomohito Izumi, Hironori Takahashi, Yohei Kawana, Kei Takahashi, Shinjiro Kodama, Keizo Kaneko, Junhong Gao, Kenji Uno, Shojiro Sawada, Tomoichiro Asano, Vladimir V. Kalinichenko, Etsuo A. Susaki, Makoto Kanzaki, Hiroki R. Ueda, Yasushi Ishigaki, Tetsuya Yamada, Hideki Katagiri

**Affiliations:** 1 Department of Metabolism and Diabetes, Tohoku University Graduate School of Medicine, Sendai, 980-8575 Japan; 2 Department of Medical Science, Graduate School of Medicine, University of Hiroshima, Hiroshima, 734-8553 Japan; 3 Division of Pulmonary Biology, Cincinnati Children’s Hospital Medical Center, Cincinnati, OH 45229 USA; 4 Department of Systems Pharmacology, Graduate School of Medicine, The University of Tokyo, Tokyo, 113-0033 Japan; 5 Laboratory for Synthetic Biology, RIKEN Quantitative Biology Center, Osaka, 565-0871 Japan; 6 PRESTO, Japan Science and Technology Agency (JST), Kawaguchi, 332-0012 Japan; 7 Tohoku University Graduate School of Biomedical Engineering, Sendai, 980-8579 Japan; 8 Japan Agency for Medical Research and Development, Project for Elucidating and Controlling Mechanisms of Aging and Longevity, Tokyo, 100-0004 Japan; 9 Japan Agency for Medical Research and Development, CREST, Tokyo, 100-1004 Japan; 10 Division of Diabetes and Metabolism, Department of Internal Medicine, Iwate Medical University, Morioka, 020-8505 Japan

## Abstract

Under insulin-resistant conditions such as obesity, pancreatic β-cells proliferate to prevent blood glucose elevations. A liver–brain–pancreas neuronal relay plays an important role in this process. Here, we show the molecular mechanism underlying this compensatory β-cell proliferation. We identify FoxM1 activation in islets from neuronal relay-stimulated mice. Blockade of this relay, including vagotomy, inhibits obesity-induced activation of the β-cell FoxM1 pathway and suppresses β-cell expansion. Inducible β-cell-specific FoxM1 deficiency also blocks compensatory β-cell proliferation. In isolated islets, carbachol and PACAP/VIP synergistically promote β-cell proliferation through a FoxM1-dependent mechanism. These findings indicate that vagal nerves that release several neurotransmitters may allow simultaneous activation of multiple pathways in β-cells selectively, thereby efficiently promoting β-cell proliferation and maintaining glucose homeostasis during obesity development. This neuronal signal-mediated mechanism holds potential for developing novel approaches to regenerating pancreatic β-cells.

## Abstract (web summary)

Neuronal signals, in particular those transmitted via the vagal nerve, regulate both β-cell function and proliferation. Here, Yamamoto et al. show that the forkhead box M1 pathway is required for vagal signal-mediated induction of β-cell proliferation during obesity.

## Introduction

Several lines of evidence indicate that terminally differentiated pancreatic β-cells retain significant proliferative capacity in vivo^1–4^ and this proliferative capacity has attracted considerable research attention in terms of both elucidating the mechanism underlying the maintenance of glucose homeostasis and developing therapeutic strategies for diabetes mellitus. From the viewpoint of maintaining glucose homeostasis, promotion of pancreatic β-cell proliferation is known to occur in insulin-resistant states, such as during obesity development, resulting in secretion of more insulin in response to increased systemic insulin demand^5^. Thus the compensatory β-cell responses appear to be an endogenous preventive mechanism that acts against diabetes development. However, the mechanism(s) by which obesity induces compensatory β-cell responses is not fully understood. It was previously reported that glucose^6^ can serve as a regulator of β-cell proliferation in these processes. However, obese humans^7^ and rodents^8^ reportedly exhibit compensatory β-cell responses prior to the onset of detectable hyperglycemia, indicating the involvement of unknown triggers, other than glucose, in these processes.

Neuronal signals, especially those transmitted via the vagal nerves, are known to be regulators of both the functions^9, 10^ and the proliferation^11^ of β-cells. In addition, several studies have suggested that vagal nerve signals are involved in compensatory β-cell proliferation in various animal models^12, 13^. However, the mechanisms, including the triggers which initiate the process of vagal nerve-induced proliferation of β-cells, have yet to be clarified.

**Fig. 1:**
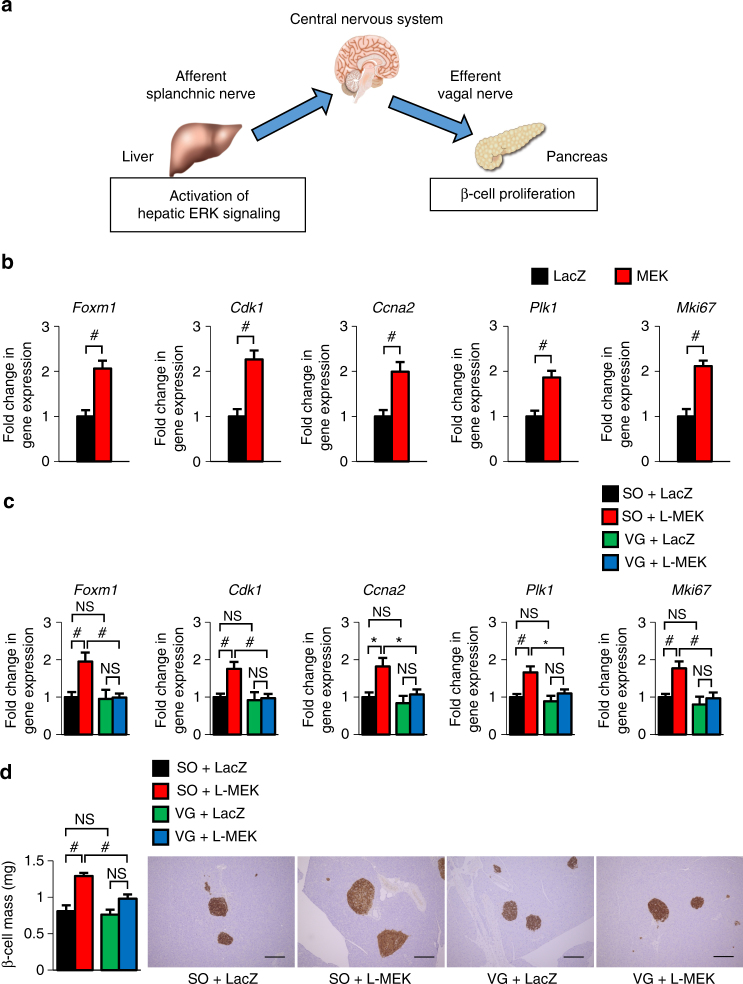
Hepatic ERK activation increases the expression levels of Foxm1 and its target genes as well as that of the Mki67 gene in islets and induces β-cell proliferation via vagal nerves. **a** Schematic model of the neuronal relay system from the liver to the pancreas. **b** Expression levels of Foxm1 and its target genes as well as that of the Mki67 gene in islets of L-MEK-mice or LacZ-injected mice on day 3 after adenoviral administration. **c** Expression levels of Foxm1 and its target genes as well as that of the Mki67 gene in islets of L-MEK-mice or LacZ-injected mice on day 3 after adenoviral administration, after vagotomy (VG), or sham operation (SO). **d** β-Cell mass in L-MEK-mice or LacZ-injected mice on day 14 after adenoviral administration, after VG, or SO; representative images are shown in the right four panels. Gene expression levels in islets or β-cell mass of LacZ-injected mice after SO served as controls **c**, **d**. Scale bar, 200 µm. Data are presented as means ± s.e.m. NS, not significant, *P < 0.05, ^#^ *P* < 0.01, assessed by unpaired *t*-test **b** or one-way ANOVA **c**, **d**. *n* = 7 or 8 **b**, *n* = 6–11 **c**, *n* = 7–11 **d**

In tackling this pivotal issue, we previously proposed an important role of neuronal signals from the liver in β-cell proliferation during obesity development^14^. Using the adenoviral gene transduction system, the active mutant of mitogen-activated protein kinase/extracellular signal-regulated kinase (ERK) kinase1 (MEK-1) was expressed in the livers of mice (L-MEK-mice), leading to hepatic ERK activation. The hepatic ERK activation was found to induce marked β-cell proliferation. This β-cell proliferation was blocked by pharmacological deafferentation of the splanchnic nerve, midbrain transection, or bilateral subdiaphragmatic dissection of the vagus nerves including those innervating the pancreas (vagotomy), demonstrating involvement of the liver–brain–pancreas neuronal relay triggered by hepatic ERK activation^14^. In addition, the ERK pathway is activated in the livers of several murine models of obesity. Blockade of this inter-organ system was found to suppress obesity-induced increases in pancreatic β-cells^14^. Thus the neuronal relay system from the liver to the pancreas (Fig. 1a) plays an important role in compensatory proliferation of pancreatic β-cells in obesity settings. However, the molecular mechanism, especially which pathway(s) in pancreatic β-cells are involved and the molecule(s) from the vagal nerve that trigger β-cell proliferation, remain to be clarified. In this study, we discover that vagal signals activate the forkhead box M1 (FoxM1) pathway in β-cells, resulting in compensatory β-cell proliferation. Furthermore, several neurotransmitters are found to efficiently activate the β-cell FoxM1 pathway, when islet cells are simultaneously treated with these factors. Thus vagal factors are involved in activation of the β-cell FoxM1 pathway that is a pivotal mechanism for maintaining glucose homeostasis, especially when disturbed by excessive energy intake.

## Results

### Vagus activates the FoxM1 pathway in L-MEK-mouse islets

To explore the molecular mechanism whereby neuronal signals elicit β-cell proliferation, we first performed microarray analyses of pancreatic islets obtained from L-MEK-mice. Mice administered recombinant adenovirus encoding LacZ were used as controls. Microarray data have been deposited in the ArrayExpress database at EMBL-EBI (www.ebi.ac.uk/arrayexpress) under accession number E-MTAB-5799. First, we confirmed that hepatic ERK phosphorylation was actually enhanced in L-MEK-mice (Supplementary Figs. 1a and 6a). Consistent with the enhancement of β-cell proliferation in L-MEK-mice, pathway analysis according to the parametric analysis of gene set enrichment (PAGE) method^15^ showed that the DNA replication reactome- and the cell cycle-related pathways were both markedly upregulated in the islets of L-MEK mice (Supplementary Table 1). Among significantly changed genes in the microarray analysis, we found expression of the *Foxm1* gene to be significantly increased in the islets of L-MEK-mice. FoxM1 affects multiple steps in the cell cycle^16^ by regulating several mitogenic genes, including cyclin-dependent kinase 1 (*Cdk1*)^17, 18^, cyclin A (*Ccna*)^18–20^, and polo-like kinase 1 (*Plk1*)^21^. Indeed, we confirmed upregulations of these FoxM1-related genes, including *Foxm1*, *Cdk1*, *Ccna2*, and *Plk1*, in islets of L-MEK-mice by quantitative reverse transcriptase-PCR (RT-PCR; Fig. 1b), indicating the FoxM1 pathway to be activated in islet cells of L-MEK-mice. Gene expression of *Mki67* encoding Ki67, a commonly used marker of cell proliferation^22^, was also increased in islets of L-MEK-mice. Importantly, the upregulations of these genes in islet cells induced by hepatic ERK activation were completely blocked by dissection of the vagus nerves including those innervating the pancreas (Fig. 1c). In addition, consistent with the results of the gene expression analysis, increases in β-cell mass in L-MEK-mice were also completely blocked by the vagotomy procedure (Fig. 1d). These findings suggest that neuronal signals, transmitted by the vagal nerves, are involved in activation of the FoxM1 pathway in β-cells and the promotion of β-cell proliferation.

### Vagus activates the FoxM1 pathway in obese mouse islets

**Fig. 2:**
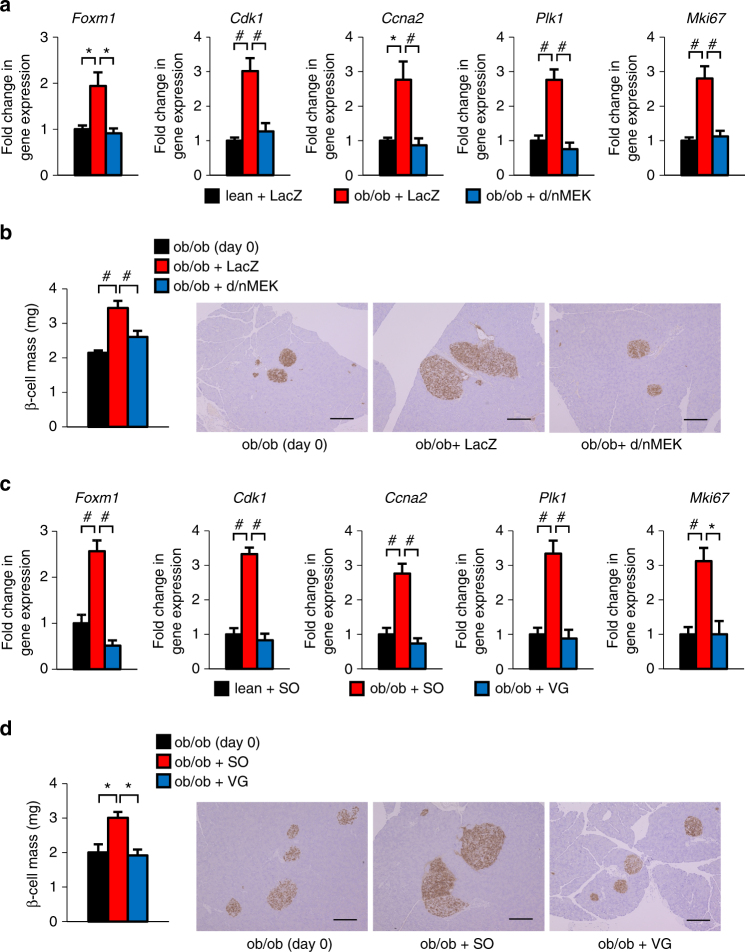
Pancreatic vagal nerves upregulate the expression levels of Foxm1 and its target genes as well as that of the Mki67 gene in islets, thereby enhancing β-cell proliferation during obesity development. **a** Expression levels of Foxm1 and its target genes as well as that of the Mki67 gene in islets of ob/ob mice on day 7 after d/nMEK or LacZ adenoviral administration. **b** β-Cell mass of 6-week-old ob/ob mice on day 7 after d/nMEK or LacZ adenoviral administration; representative images are shown in the right three panels. **c** Expression levels of Foxm1 and its target genes as well as that of the Mki67 gene in islets of ob/ob mice on day 7 after VG or SO. **d** β-Cell mass of ob/ob mice on day 7 after VG or SO; representative images are shown in the right three panels. Gene expression levels in islets of LacZ-injected- **a** or SO **c **lean mice  served as controls. β-Cell mass of 5-week-old ob/ob mice before adenoviral administration **b** or operation **d** served as controls (ob/ob (day 0)). Scale bar, 200 µm. Data are presented as means ± s.e.m. NS, not significant, ^*^ *P* < 0.05, ^#^ *P* < 0.01, assessed by one-way ANOVA. *n* = 4 **a**, *n* = 6 **b**, *n* = 4 or 6 **c**, *n* = 3–6 **d**

We next explored the significance of this mechanism in compensatory β-cell proliferation in obesity settings. As reported previously^14^, ERK phosphorylation was enhanced in the livers of ob/ob mice (Supplementary Figs. 1b and 6b). In addition, *Foxm1* and its target genes as well as *Mki67* in islets were upregulated (Supplementary Fig. 1c) in ob/ob mice. Magnitudes of hepatic ERK phosphorylation in L-MEK-mice were slightly higher than that in ob/ob mice (Supplementary Figs. 1d and 6c). To examine the causal relationship between activations of the hepatic ERK and the β-cell FoxM1 pathways during obesity development, we attempted to suppress hepatic ERK activity in the livers of ob/ob mice by expressing the dominant-negative MEK mutant (d/nMEK) employing the adenoviral gene transduction system. We confirmed that hepatic expression of d/nMEK actually suppressed hepatic ERK phosphorylation (Supplementary Figs. 1e and 6d). Interestingly, suppression of the hepatic ERK pathway blocked upregulations of *Foxm1* and its target genes, as well as that of *Mki67*, in islets of ob/ob mice (Fig. 2a). Furthermore, obesity-induced increases in β-cell mass were also blunted by inhibition of the hepatic ERK pathway (Fig. 2b). Thus activation of the hepatic ERK pathway during obesity development is involved in activation of the FoxM1 pathway in β-cells, thereby increasing β-cell mass.

We then examined whether this liver–pancreas interaction involves vagal signals. As observed in L-MEK-mice, the vagotomy procedure completely blocked obesity-induced upregulations of *Foxm1*, its target genes, and that of *Mki67* (Fig. 2c). Furthermore, increments in β-cell mass (Fig. 2d) in ob/ob mice were also blocked by vagotomy. These findings suggest that the liver–pancreas neuronal relay functioning in L-MEK-mice is also required for compensatory β-cell proliferation during obesity development. In addition, vagal nerve signals were shown to be involved in activation of the β-cell FoxM1 pathway in this process.

### FoxM1 knockout blocks vagus-mediated β-cell proliferation

**Fig. 3:**
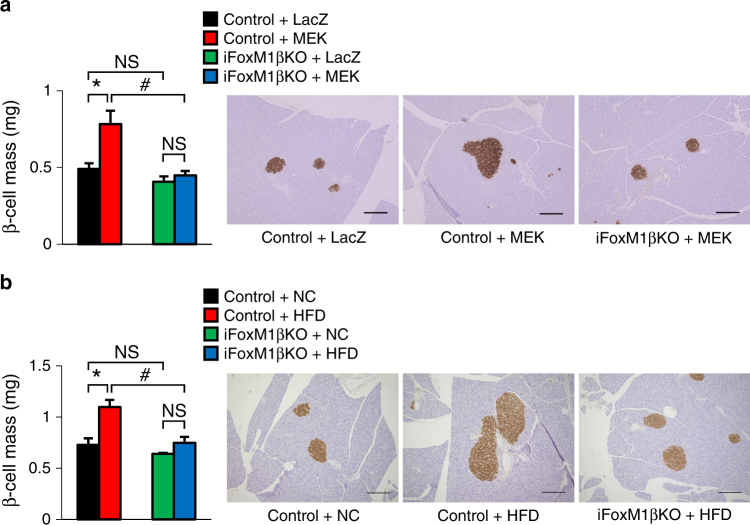
FoxM1 is essential for vagal signal-induced compensatory β-cell proliferation. **a** β-Cell mass of iFoxM1βKO or control mice on day 16 after L-MEK or LacZ administration; representative images are shown in the right three panels. **b** β-Cell mass of iFoxM1βKO or control mice fed a high-fat diet (HFD) or normal chow (NC) for 15 weeks; representative images are shown in the right three panels. Scale bar, 200 µm. Data are presented as means ± s.e.m. NS, not significant, *P < 0.05, ^#^ *P* < 0.01, assessed by one-way ANOVA. *n* = 3–6

The aforementioned findings indicate that hepatic activation of the ERK pathway induces β-cell proliferation with activation of the β-cell FoxM1 pathway involving vagal signals. To explore whether β-cell FoxM1 is actually important in this process, which is observed in both L-MEK-mice and obese mice, we generated tamoxifen-inducible β-cell-specific FoxM1 knockout mice (iFoxM1βKO mice) by crossing RIP-CreER mice^1^ and FoxM1-floxed mice^23^. Tamoxifen administration markedly decreased *Foxm1* expression in islets isolated from iFoxM1βKO mice (Supplementary Fig. 2a). Since rodent pancreatic islets reportedly consist of 80% β-cells and 20% other endocrine cells^24^, the FoxM1 gene was calculated to be deleted in 80–90% of β-cells in iFoxM1βKO mice. First, to examine the effects of β-cell FoxM1 deficiency on increases in β-cell mass induced by hepatic ERK activation, we administered the recombinant adenovirus encoding the active mutant of MEK-1. Notably, in iFoxM1βKO mice, β-cell mass failed to be increased in response to MEK-1 expression in the liver (Fig. 3a). Thus activation of the β-cell FoxM1 pathway is essential for promoting the β-cell proliferation observed in L-MEK-mice.

FoxM1 deficiency in β-cells also produced a striking phenotype in terms of obesity-related β-cell increases. For this experiment, wild-type and iFoxM1βKO mice were fed a high-fat diet (HFD). While these two groups of mice exhibited similar body weight gains after HFD loading (Supplementary Fig. 2b), increases in β-cell mass after 15-week HFD loading were significantly inhibited in the iFoxM1βKO mice (Fig. 3b). Accordingly, impaired glucose tolerance became apparent in iFoxM1βKO mice under the HFD-fed condition, whereas normal chow-fed iFoxM1βKO mice showed glucose tolerance similar to that of control mice (Supplementary Fig. 2c). Plasma insulin levels of HFD-fed iFoxM1βKO mice tended to be lower than those of HFD-fed control mice, despite higher blood glucose levels (Supplementary Fig. 2d). These findings demonstrate that β-cell FoxM1 plays a prerequisite role in maintaining glucose homeostasis, through compensatory β-cell proliferation, during obesity development.

As observed in ob/ob mice, increases in the expression levels of *Foxm1, Cdk1*, *Ccna2*, and *Plk1* as well as that of *Mki67* were observed in islets of wild-type control mice after the HFD loading (Supplementary Fig. 3a). It merits emphasis that these increases were detectable as early as 1 week after starting the HFD (Supplementary Fig. 3a), before weight gain became evident. Interestingly, at the same time point, hepatic ERK activation was also already apparent (Supplementary Figs. 3b and  6e). In contrast, upregulations of the cell cycle-related genes in islet cells were markedly blocked in iFoxM1βKO mice (Supplementary Fig. 3c). Thus FoxM1 expressed in β-cells is essential for β-cell proliferation via promotion of the cell cycle. Collectively, these results indicate that β-cell proliferation via neuronal signals is mediated by activation of the FoxM1 pathway in β-cells, which may underlie the maintenance of glucose homeostasis during obesity development.

### Multiple vagal factors enhance β-cell proliferation

**Fig. 4:**
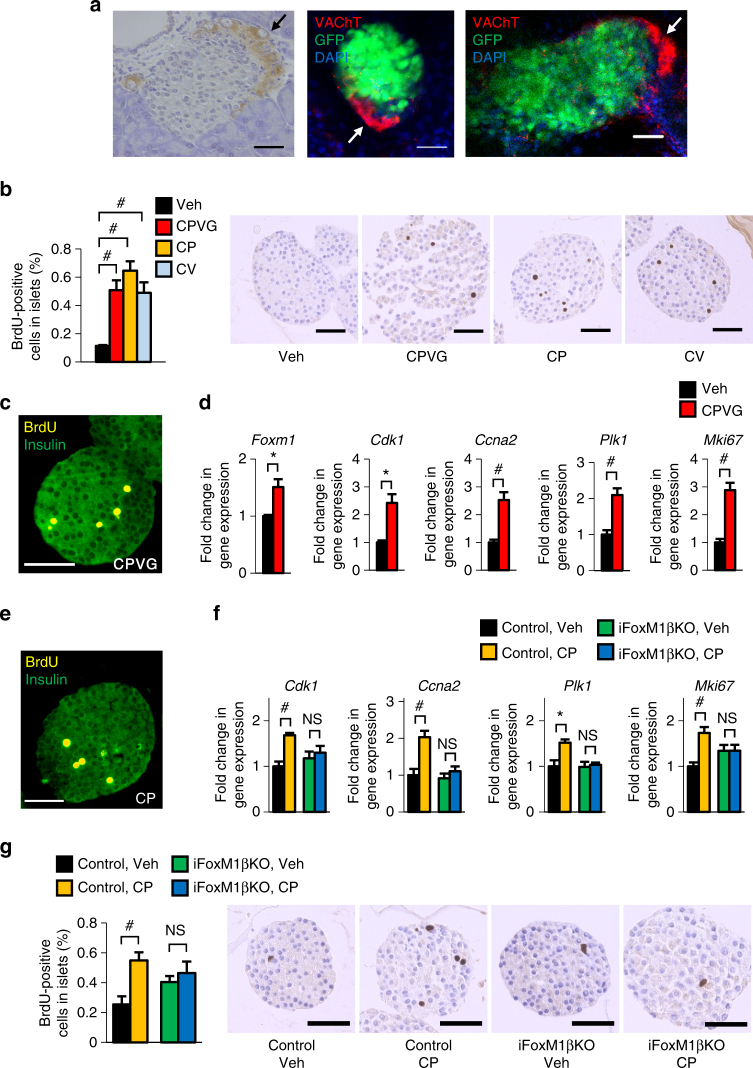
Treatments combining carbachol with either PACAP or VIP induce β-cell proliferation through a FoxM1-dependent mechanism. **a** Representative images of parasympathetic ganglia (arrows) are shown. Left: VAChT immunostaining image. Middle, right: Fluorescent immunostaining images. **b** BrdU-positive cell ratios of CPVG-, CP-, and CV-treated islets, isolated from C57BL/6 N mice, after 48 h incubation; representative images are shown in the right four panels. **c** Fluorescent immunostaining of CPVG-treated islets, isolated from C57BL/6 N mice. **d** Expression levels of Foxm1 and its target genes as well as that of the Mki67 gene in CPVG-treated islets, isolated from C57BL/6 N mice. **e** Fluorescent immunostaining of CP-treated islets, isolated from C57BL/6 N mice. **f** Expression levels of FoxM1 target genes and that of the Mki67 gene in CP-treated islets, isolated from iFoxM1βKO or control mice. **g** BrdU-positive cell ratios of CP-treated islets, isolated from iFoxM1βKO or control mice; representative images are shown in the right four panels. BrdU-positive cell ratios and gene expression levels of Veh-treated islets served as controls **b**, **d**, **f**, **g**. Scale bar, 50 µm. Data are presented as means ± s.e.m. NS, not significant, *P < 0.05, ^#^ *P* < 0.01, assessed by unpaired *t*-test **b**, **d**, **f**, **g**. *n* = 5 **b**, **d**, *n* = 4–6 **f**, *n* = 10–12 **g**

The vagal nerves innervating pancreatic islets reportedly terminate at parasympathetic ganglia^25^. Employing the tissue clearing method, CUBIC^26^, we made the intriguing observation that parasympathetic ganglia were mainly located in the vicinity of islets (Fig. 4a). This anatomical structure may allow the vagus to selectively and strongly stimulate β-cells. Therefore, we next attempted to elucidate the molecular mechanism(s) by which signals from the postganglionic vagal nerve in the pancreas promote activation of the FoxM1 pathway and the proliferation of β-cells.

Postganglionic neurons reportedly express several neuropeptides, including pituitary adenylate cyclase activating polypeptide (PACAP), vasoactive intestinal polypeptide (VIP) and gastrin-releasing peptide (GRP), in addition to acetylcholine^27–30^. Therefore, we first treated pancreatic islets, isolated from mice, with a combination of PACAP, VIP, GRP, and carbachol, a cholinergic receptor agonist. We used these reagents at the same concentrations as those at which they are usually used for in vitro experiments and are known to enhance glucose-stimulated insulin secretion from isolated islets or β-cell lines^31–33^. Histological analyses revealed that bromodeoxyuridine (BrdU)-positive cells were increased in islets treated when all four reagents were administered for 48 h in combination: carbachol, PACAP, VIP, and GRP (CPVG), by 4.5-fold (Fig. 4b), with nearly all of the BrdU-positive cells also being positive for insulin (Fig. 4c). These findings indicate that these transmitters per se have the capacity to directly promote β-cell proliferation. We thus developed an ex vivo experimental system allowing us to analyze β-cell-proliferative capacity. Interestingly, expression levels of FoxM1-related genes were significantly increased in CPVG-treated islets (Fig. 4d), suggesting that these neurotransmitters promote β-cell proliferation via activation of the FoxM1 pathway.

### Carbachol plus PACAP/VIP increases β-cells through FoxM1

Next, to determine which of these four factors is critical in the process of promoting β-cell proliferation, we examined the effects of individual factors on β-cell proliferation by examining the expression levels of FoxM1 target genes and that of the *Mki67* gene in islets. Since massive amounts of pancreatic islets were required for these experiments, we utilized islets isolated from rats. First, we confirmed CPVG-induced upregulations of the FoxM1 target genes and the *Mki67* gene in rat islets (Supplementary Fig. 4a). In contrast, treatment with each factor alone did not upregulate these genes in a fashion similar to that observed with CPVG (Supplementary Fig. 4a). Therefore, we next examined the effects of withdrawal of each of the four factors individually. Notably, unlike the other three neuropeptides, withdrawal of carbachol blunted the upregulations of these gene expression levels such that the differences were no longer statistically significant, suggesting an essential role of carbachol in promoting β-cell proliferation (Supplementary Fig. 4a).

We then examined the effects of stimulating rat isolated islets with carbachol combined with other neuropeptides. Addition of GRP to other neurotransmitters did not result in significant effects on expression levels of either the FoxM1 target genes or that of the *Mki67* gene, indicating a minimal role of GRP. In contrast, among several combinations of carbachol and neuropeptides, carbachol plus PACAP (CP) treatment maximally increased the expression levels of the FoxM1 target genes and that of the *Mki67* gene in a fashion similar to that observed with CPVG (Supplementary Fig. 4a). Importantly, the effects on *Cdk1* expression of CP treatment exceeded the maximal increments induced by either C or P alone (Supplementary Fig. 4b). Thus simultaneous stimulation with these two factors appears to be needed to activate the FoxM1 pathway.

Addition of VIP to carbachol (CV) also significantly increased *Cdk1* expression in rat islets. Therefore, we further examined the effects of CP or CV on increases in FoxM1 target genes using murine isolated islet cells. Both CP and CV significantly increased FoxM1 target gene and *Mki67* gene expression levels in murine islets, with increases similar to those produced by CPVG treatment (Supplementary Fig. 4c). We next performed BrdU staining of murine islets treated with CP or CV and obtained results indicating that these treatments significantly increased BrdU-positive islet cells by 5.7- and 4.3-fold, respectively, extents similar to those of CPVG treatment (Fig. 4b). Further histological analysis showed that nearly all BrdU-positive cells were also positive for insulin in CP-treated islets (Fig. 4e). Interestingly, the expression levels of CDK inhibitors, such as *Cdkn2b (p15Ink4b)* and *Cdkn1b (p27kip1)* were significantly and tended to be decreased, respectively, in CP-treated islets (Supplementary Fig. 4d). Thus carbachol, when combined with either PACAP or VIP, apparently exerts a synergistic effect on the induction of cell cycle-related genes, resulting in marked proliferation of β-cells in this experimental setting. On the other hand, addition of VIP to CP treatment did not further upregulate FoxM1 target genes (Supplementary Fig. 4a). Thus, when added to carbachol, either PACAP or VIP can exert sufficient β-cell proliferation-promoting effects. Furthermore, PACAP and VIP are equivalent in this regard and thus interchangeable.

Finally, we examined the effects of CP treatment on β-cell proliferation using islets isolated from iFoxM1βKO mice. Upregulations of the FoxM1 target genes and the *Mki67* gene were markedly blunted in CP-treated islets isolated from these mice (Fig. 4f). Furthermore, increases in BrdU-positive cells in response to CP treatment were completely abolished in islets isolated from iFoxM1βKO mice (Fig. 4g). Thus the combined treatment promotes β-cell proliferation through a FoxM1-dependent mechanism (Supplementary Fig. 5).

## Discussion

We herein clarified that the liver–brain–pancreas neuronal relay activates β-cell FoxM1, leading to β-cell proliferation. This mechanism, which is involved in compensatory β-cell proliferation during obesity development, consists of hepatic ERK activation, transmission via a neuronal relay, and FoxM1-dependent cell cycle promotion in β-cells (Supplementary Fig. 5). In addition, vagal factors were identified as serving as activators of the β-cell FoxM1 pathway. Anatomical and molecular findings strongly suggest the advantage of neuronal signals in achieving high concentrations of multiple factors simultaneously around β-cells locally. Thus, based on the present findings, we propose that the neuronal system itself can achieve selective and strong transmission of multiple vagal signals to islet cells, leading to efficient β-cell proliferation.

We began the present study by comprehensively examining mRNA expression levels in pancreatic islet cells from L-MEK-mice and found FoxM1 and its downstream molecules to be upregulated. Several lines of evidence have recently highlighted the importance of FoxM1 in β-cell mass regulation. First, FoxM1 deficiency in the entire pancreas induces a gradual decline in β-cell mass with age^34^. In addition, proliferation of β-cells after partial pancreatectomy^35^ and during pregnancy^36^ is blunted in the pancreas-specific FoxM1 knockout mice. Furthermore, FoxM1 in pancreatic islets is reportedly upregulated in obese mice^37^. These findings indicate significant roles of FoxM1 in β-cell proliferation. However, there have been no reports on the effects of β-cell-specific FoxM1 deficiency. In particular, whether FoxM1 upregulation is involved in obesity-induced β-cell proliferation and, if so, how β-cell FoxM1 is activated, have yet to be determined. Most notably, extracellular factors, which are potentially involved, remain largely unknown. First, we observed that FoxM1 was upregulated in islets of obesity model mice. The vagotomy procedure blocked β-cell FoxM1 activation and β-cell increases in both L-MEK-mice and obesity model mice. In addition, using isolated islets, vagal factors were shown to actually activate the FoxM1 pathway and to trigger proliferation of β-cells. Thus vagal signals are involved in activation of the β-cell FoxM1 pathway. Furthermore, inducible β-cell-specific FoxM1 deficiency blocked β-cell proliferation, both that induced by HFD in vivo and that induced by vagal factors ex vivo. Thus the vagal factors-β-cell FoxM1 pathway plays prerequisite roles in promoting β-cell proliferation and maintaining glucose homeostasis during obesity development.

Notably, hepatic ERK activation and marked increases in the expression levels of FoxM1 and its target genes as well as that of the *Mki67* gene were observed as early as only 1 week after HFD loading (Supplementary Fig. 3a, b) when obesity had not yet become evident. It was recently reported that HFD loading for even short periods makes the gut “leaky”, allowing the entry of pro-inflammatory factors into the portal vein, thereby triggering the development of insulin resistance^38^. This mechanism might be involved in activation of the hepatic ERK pathway after short-duration HFD feeding. Of note, β-cell FoxM1 deficiency completely suppressed HFD-induced upregulations of both cell cycle-related genes and the *Mki67* gene (Supplementary Fig. 3c). Thus hepatic sensing of inflammatory signals and transmission of neuronal signals likely contribute to maintaining glucose homeostasis, providing protection from anticipated insulin resistance in the very early phase of obesity development, by promoting compensatory β-cell proliferation via β-cell FoxM1 upregulation/activation.

Anatomically, vagal nerves originate in the dorsal motor nucleus of the vagus in the brainstem, and preganglionic vagal fibers directly project to peripheral organs. In the pancreas, all preganglionic vagal fibers reportedly terminate at pancreatic parasympathetic ganglia, resulting in transmission to postganglionic neurons^25, 39^. These anatomical findings suggest that neuronal signals which elicit β-cell proliferation are produced in pancreatic parasympathetic ganglionic cells. A few prior reports described observational findings indicating that some of the parasympathetic ganglia are located in the vicinity of pancreatic islets. In the present study, three-dimensional observation of the CUBIC-mediated cleared pancreatic samples enabled us to obtain precise information from stereo images and to thereby determine the locations of pancreatic parasympathetic ganglia. Interestingly, we observed parasympathetic ganglia to be mainly located adjacent to pancreatic islets, suggesting selective and strong transmission of vagal signals to islet cells, especially β-cells, within the pancreas.

Furthermore, combined treatment with carbachol, an acetylcholine mimetic, and either PACAP or VIP synergistically induced β-cell proliferation. Acetylcholine exerts its insulinotropic effects on β-cells through the muscarinic 3 (M3) receptor, a Gq-linked G protein-coupled receptor (GPCR)^40^. It was previously reported that, using mice which express a Gq-coupled designer GPCR selectively in β-cells, activation of Gq signaling is involved in β-cell proliferation^41^. GRP also reportedly stimulates insulin secretion through the GRP receptor, a Gq-linked GPCR^42, 43^. However, in the present study, in contrast to carbachol, GRP exerted minimal effects on upregulations of cell cycle-related genes and β-cell proliferation even when combined with PACAP (Supplementary Fig. 4a). This might be attributable to differences in downstream molecules between M3 and GRP receptors^27, 42, 44^. On the other hand, PACAP reportedly induces insulin secretion from β-cells, possibly through VPAC2 and PAC1 receptors^32^, both of which are Gs-linked GPCR^45^. The Gs-coupled VPAC2 receptor is also shared by VIP^46^ and VIP was reported to induce insulin secretion from β-cells through this receptor^47^. It is well known that activation of Gs signaling by glucagon like peptide-1 enhances β-cell proliferation^48^. The results obtained in this study show that combining acetylcholine, a Gq-signaling activator, with PACAP or VIP, both of which are Gs-signaling activators, exerts synergistic impacts on activation of the FoxM1 pathway (Supplementary Fig. 5), although further investigations are needed to elucidate the molecular mechanism underlying the process from the GPCRs to the FoxM1 pathway. Thus, taking advantage of the vagal system in the pancreas, which releases multiple neurotransmitters and achieves high concentrations of these factors locally around β-cells, the Gq- and Gs-signaling pathways in β-cells can be stimulated simultaneously, leading to efficient β-cell proliferation and thereby to the maintenance of proper glucose metabolism.

As shown in Fig. 4f, g, BrdU-positive cells and Mki67 expression in pancreatic islets isolated from iFoxM1βKO mice were slightly increased as compared with those from control mice. In contrast, FoxM1 deficiency did not affect the expression levels of FoxM1-related cell cycle genes in isolated islet cells (Fig. 4f). These findings suggest that FoxM1 deficiency in β-cells induces compensatory activation of a FoxM1-independent, growth-promoting pathway under isolated condition. However, as shown in Fig. 3b, β-cell mass of normal chow-fed control and iFoxM1KO mice were similar, indicating that the putative FoxM1-independent pathway is insignificant in vivo. In addition, CP treatment neither upregulated *Cdk1*,* Ccna2*, and *Plk1* expression levels nor further stimulated FoxM1 KO islet cell proliferation (Fig. 4f, g). Therefore, these findings indicate that a FoxM1-dependent mechanism mediates the proliferative effects of simultaneous stimulation of Gs and Gq signaling by vagal factors.

In human islet cells, several cell cycle inhibitors, such as cyclin-dependent kinase (CDK) inhibitors, are abundantly expressed, and this is speculated to be one of the causes of low responsiveness of human islets cells to several proliferation stimulants^49^. Indeed, promotion of human β-cell proliferation by adenovirus-mediated silencing of p27 along with overexpression of both CDK6 and cyclin D3 were recently reported^50^. In the present study, treatment with vagal factors decreased the expression levels of CDK inhibitors, such as *Cdkn1b* and *Cdkn2b*, in mouse islets (Supplementary Fig. 4d). In addition, *Cdkn1b* was reported to be a FoxM1 target gene^16^. Therefore, vagal signals can suppress CDK inhibitors, concomitantly with increasing several cell cycle-accelerating molecules, through FoxM1 activation. Further studies, including experiments designed to examine whether the vagal factors actually suppress CDK inhibitors and promote proliferation in human islets, are needed for clinical application of these vagal factors.

Impairment of limb regeneration^51, 52^ and retardation of liver regeneration^53^ due to surgical denervation are widely recognized. Thus tissue regeneration regulated by neuronal signals has already been advocated for a few decades. However, the molecular mechanisms underlying these phenomena remain a mystery. As we have shown herein, vagal nerve-derived factors induce β-cell proliferation. Taking a broader perspective, the concept of neuronal factors regulating cell cycle-related genes and promoting cell proliferation may open a new avenue of research in the field of tissue regeneration.

## Methods

### Animals

Animal studies were conducted in accordance with the Tohoku University institutional guidelines. Ethical approval has been obtained from the Institutional Animal Care and Use Committee of the Tohoku Univesity Environmental&Safety Committee. Eight-week-old male C57BL/6 N mice and 10-week-old male Sprague-Dawley (SD) rats were purchased from Japan SLC (Shizuoka, Japan). Five-week-old homozygous male C57BL/6J-Lep^ob^ (ob/ob) mice and their male lean littermates (+/?) were purchased from Charles River Laboratories Japan (Yokohama, Japan). For ob/ob or lean mice, islet isolation or extraction of the pancreas was performed at 6 weeks of age, 7 days after adenoviral administration, or vagotomy. The rat insulin 2 promoter-CreER (RIP-CreER) mice^1^ and MIP-GFP mice^54^ were purchased from The Jackson Laboratory (Bar Harbor, Maine, USA). To obtain tamoxifen-inducible β-cell-specific FoxM1 knockout (iFoxM1βKO) mice, we crossed RIP-CreER mice and FoxM1^flox/flox^ mice. At 8 weeks of age, male RIP-CreER; FoxM1^flox/flox^ mice and male FoxM1^flox/flox^ mice (as controls) were injected intraperitoneally with tamoxifen (Sigma, St. Louis, MO, USA), at 80 µg/g body weight, dissolved in corn oil (Sigma) every 24 h for 5 consecutive days. At 7 days after completion of the tamoxifen injections, HFD loading was started, the adenovirus was administered, or islet isolation was performed. Mice with HFD-induced obesity were produced by high-fat chow (32% safflower oil, 33.1% casein, 17.6% sucrose, and 5.6% cellulose) feeding^55^, beginning at 8 weeks of age. These animals were housed in an air-conditioned environment, with a 12-h light–dark cycle. Only male mice were used for the experiments.

### Recombinant adenovirus

Recombinant adenoviruses were propagated in HEK293 cells and purified by cesium chloride gradient ultracentrifugation. Titers of virus stock were determined by end point cytopathic effect assay^14^. Then, 1 × 10^8^ plaque-forming units (PFU) per mouse of the adenovirus encoding the constitutively active mutant of the Xenopus MEK1 gene for hepatic ERK activation (L-MEK) and 5 × 10^8^ PFU per mouse of the adenovirus encoding the dominant-negative mutant of the mouse MEK1 gene for hepatic ERK suppression (d/nMEK) were intravenously injected. The controls received 1 × 10^8^ PFU per mouse of LacZ adenovirus.

### Immunoblotting

Liver samples were homogenized in ice-cold lysis buffer containing 50 mM Tris, pH 7.4, 100 mM NaCl, 10 mM EDTA, 10% glycerol, 1% Nonidet P-40, 1 mM sodium orthovanadate, 2 mM phenylmethylsulfonyl fluoride, 40 mM β-glycerophosphate, 50 mM NaF, 2 µg/ml aprotinin, and 2 µg/ml leupeptin. Tissue homogenates were centrifuged at 13,500×*g*. Supernatants including tissue protein extracts (100 µg total protein) were boiled in Laemmli buffer containing 10 mM dithiothreitol, subjected to sodium dodecyl sulfate-polyacrylamide gel electrophoresis, and transferred onto nitrocellulose membranes. The membranes were incubated with antibodies to ERK (#9102, Cell Signaling Technology, Danvers, MA, USA) at 1:5000 dilution, phospho-ERK (#4376, Cell Signaling Technology) at 1:5000 dilution, or actin (A2066, Sigma) at 1:2000 dilution, and then incubated with a secondary horseradish peroxidase-conjugated antibody (NA9340, GE Healthcare, Tokyo, Japan) at 1:10,000 dilution. The immunoblots were visualized with Pierce ECL Plus Western Blotting Substrate (Thermo Fisher Scientific, Yokohama, Japan).

### Vagotomy

A laparotomy incision was made on the ventral midline. For subdiaphragmatic vagotomy, both the ventral and dorsal vagal trunks were separated from the esophagus, and both the ventral vagal trunk below the bifurcation of the hepatic branch and the dorsal vagal trunk were transected^11, 13, 14^. The sham operation was performed using an identical procedure but with the nerves left intact. At 7 days after these operations, adenovirus administration, islet isolation, or extraction of the pancreas was performed.

### Islet isolation

Pancreatic islets were isolated by retrograde infusion of 1.0 ml cold Hanks’ balanced salt solution containing 1.0 mg/ml collagenase V (Sigma) into the pancreatic duct. Pancreata were digested in a thermostat chamber at 37 °C. The islets from mice were purified by hand-picking under a light microscopic view^56^. The islets from rats were separated by density gradient centrifugation using Ficoll (GE Healthcare, Tokyo, Japan)–Conray (Daiichi-Sankyo, Tokyo, Japan) solution^57^ and purified by hand-picking under a light microscopic view.

### Microarray analysis

Total RNA was extracted using an RNeasy Micro Kit (QIAGEN) from isolated mouse islets. Cy3-Labeld cRNA was synthesized employing the Agilent Low Input Quick Amp Labeling Kit, one color (Agilent Technologies, Santa Clara, CA, USA). After hybridization of cRNA samples with a Gene Expression Hybridization Kit (Agilent), the microarray slide was scanned on a DNA microarray scanner (Agilent). Image data were processed by Feature Extraction 10.7.3.1 (Agilent). Data analyses were performed using the GeneSpring software GX12.1 (Agilent). Pathway analysis was performed according to the PAGE method^15^. Microarray data have been deposited in the ArrayExpress database at EMBL-EBI (www.ebi.ac.uk/arrayexpress) under accession number E-MTAB-5799.

### Evaluation of gene expression levels by quantitative RT-PCR

Total RNA was extracted using an RNeasy Micro Kit (QIAGEN) from islets. cDNA synthesized from 100 ng of total RNA with a QuantiTect Reverse Transcription Kit (QIAGEN) was evaluated with a real-time PCR quantitative system (Light Cycler software; Roche Diagnostics, Mannheim, Germany)^58^. Relative amounts of mRNA were calculated with *Actb* mRNA as the invariant control. The sequences of primers used are listed in Supplemental Table 2.

### Measurement of β-cell mass

Pancreata were fixed with 10% formalin, minced with a razor, and embedded in paraffin. The sections were immunostained with monoclonal anti-insulin antibody (I2018, Sigma). Immunoreactivity was visualized by incubation with a substrate solution containing 3,3′-diaminobenzidine tetrahydrochloride (DAB). The tissue samples were sectioned at an interval of 100 µm and the ratio of insulin-positive areas to total pancreatic tissues was determined using a microscope, the BIOREVO BZ-9000 (Keyence, Osaka, Japan), and a BZ-2 Analyzer (Keyence). β-Cell mass was calculated by multiplying the ratio of insulin-positive areas by pancreatic weight.

### Glucose tolerance tests

Glucose tolerance tests were performed on mice fasted for 10 h. Mice were intraperitoneally injected 2 g/kg glucose, followed by measurement of blood glucose^56^. Plasma insulin levels were measured using a Mouse Insulin ELISA Kit (Morinaga, Tokyo, Japan).

### Immunostaining and tissue clearing method

Pancreata were extracted, fixed with 10% formalin, and embedded in paraffin. Sections were stained with hematoxylin and eosin and then immunostained with VAChT polyclonal rabbit antibody (139103, Synaptic Systems, Goettingen, Germany) at 1:50 dilution. Immunoreactivity was visualized by incubation with a substrate solution containing 3,3′-DAB. For fluorescent immunostaining, MIP-GFP mice were anesthetized, perfusion fixed with 4% paraformaldehyde, and cleared employing the CUBIC protocol^26^. Transparentized pancreata were stained with 4′, 6-diamidino-2-phenylindole (043-18804, Wako, Osaka, Japan), and then immunostained with VAChT polyclonal rabbit antibody (139103, Synaptic Systems) at 1:50 dilution as the primary antibody and Alexa Fluor 594 goat anti-rabbit IgG (A11037, Invitrogen) at 1:750 dilution as the secondary antibody. Immunostained transparentized samples were examined with multi-photon confocal microscope A1R-MP + (Nikon, Tokyo, Japan) and NIS elements AR (Nikon).

### Islet studies

Isolated islets were maintained overnight at 37 °C with 5% CO_2_ and 95% air in RPMI1640 medium containing 10% fetal calf serum, 25 mM glucose, 100 U/ml penicillin, 100 µg/ml streptomycin, and 50 µg/ml gentamycin. The next day, isolated islets were treated with 1.0 mg/ml collagenase for 10 min at 37 °C to assure that neuropeptides reached the inner portions of the islets^59^, washed twice with phosphate buffered saline, and then incubated for 48 h in RPMI1640 medium containing 5.5 mM glucose and 1 mM Diprotin A (Peptide Institute, Osaka, Japan), a dipeptidyl peptidase-4 inhibitor, with vagal neuropeptides. Then 100 µM of the cholinergic agonist carbachol (Nacalai Tesque, Kyoto, Japan)^31^, 100 nM PACAP -27 (Bachem, Weil am Rhein, Germany)^32^, 100 nM VIP (Sigma)^32^, and 100 nM GRP (Sigma)^33^ were used in several different combinations. Water was used as the vehicle.

### BrdU in situ detection

Isolated islets were incubated with 10 µM BrdU during the last 24 h according to the manufacturer’s protocol. At the completion of incubation, the islets were collected in a collodion bag consisting of the inner wall surfaces of tubes, fixed with 10 % formalin, and embedded in paraffin^60^. Islet sections were made at an interval of 20 µm and the labeled cells were immunostained with biotinylated anti-BrdU antibody (51–75512 L, BD Bioscience, San Jose, CA, USA) according to the BrdU In Situ Detection Kit protocol (BD Bioscience). Immunoreactivity was visualized by incubation with a substrate solution containing 3,3′-DAB. For analysis, approximately 3000–8000 nuclei of islets were counted. For fluorescent immunostaining, monoclonal anti-insulin antibody (I2018, Sigma) and biotinylated anti-BrdU antibody (51–75512 L, BD Bioscience) were used as primary antibodies, and Alexa Fluor 488 goat anti-mouse IgG1 (A21121, Invitrogen, Carlsbad, CA, USA) and Alexa Fluor 546 conjugate (S11225, Invitrogen) were used as secondary antibodies.

### Statistical analysis

No randomization was used in this study, and the investigators were not blinded to group allocation during the experiments or to the outcome assessments. No statistical method was used to predetermine sample size. Most sample sizes were chosen based on data shown in previous publications. All data were expressed as means ± s.e.m. The statistical significance of differences between two groups was assessed employing the unpaired *t*-test. For experiments involving three or more groups, one-way analysis of variance followed by Bonferroni’s post hoc test was used.

### Data availability

The microarray data have been deposited in the ArrayExpress at EMBL-EBI (www.ebi.ac.uk/arrayexpress) under accession number E-MTAB-5799. The data that support the findings of this study are available from the corresponding author upon reasonable request.

## Electronic supplementary material

Supplementary Information

## References

[1] Dor Y, Brown J, Martinez OI, Melton DA (2004). Adult pancreatic beta-cells are formed by self-duplication rather than stem-cell differentiation. Nature.

[2] Georgia S, Bhushan A (2004). Beta cell replication is the primary mechanism for maintaining postnatal beta cell mass. J. Clin. Invest..

[3] Nir T, Melton DA, Dor Y (2007). Recovery from diabetes in mice by beta cell regeneration. J. Clin. Invest..

[4] Meier JJ (2008). Beta-cell replication is the primary mechanism subserving the postnatal expansion of beta-cell mass in humans. Diabetes.

[5] Prentki M, Nolan CJ (2006). Islet beta cell failure in type 2 diabetes. J. Clin. Invest..

[6] Terauchi Y (2007). Glucokinase and IRS-2 are required for compensatory beta cell hyperplasia in response to high-fat diet-induced insulin resistance. J. Clin. Invest..

[7] Utzschneider KM (2006). Impact of differences in fasting glucose and glucose tolerance on the hyperbolic relationship between insulin sensitivity and insulin responses. Diabetes Care.

[8] Chick WL, Like AA (1970). Studies in the diabetic mutant mouse. 3. Physiological factors associated with alterations in beta cell proliferation. Diabetologia.

[9] Kaneto A, Kosaka K, Nakao K (1967). Effects of stimulation of the vagus nerve on insulin secretion. Endocrinology.

[10] Niijima A (1989). Nervous regulation of metabolism. Prog. Neurobiol..

[11] Kiba T (1996). Ventromedial hypothalamic lesion-induced vagal hyperactivity stimulates rat pancreatic cell proliferation. Gastroenterology.

[12] Lausier J (2010). Vagal control of pancreatic ss-cell proliferation. Am. J. Physiol. Endocrinol. Metab..

[13] Edvell A, Lindstrom P (1998). Vagotomy in young obese hyperglycemic mice: effects on syndrome development and islet proliferation. Am. J. Physiol..

[14] Imai J (2008). Regulation of pancreatic {beta} cell mass by neuronal signals from the liver. Science.

[15] Kim SY, Volsky DJ (2005). PAGE: parametric analysis of gene set enrichment. BMC Bioinformatics.

[16] Wierstra I (2013). The transcription factor FOXM1 (Forkhead box M1): proliferation-specific expression, transcription factor function, target genes, mouse models, and normal biological roles. Adv. Cancer Res..

[17] Zhao YY (2006). Endothelial cell-restricted disruption of FoxM1 impairs endothelial repair following LPS-induced vascular injury. J. Clin. Invest..

[18] Wang X (2002). Increased hepatic Forkhead Box M1B (FoxM1B) levels in old-aged mice stimulated liver regeneration through diminished p27Kip1 protein levels and increased Cdc25B expression. J. Biol. Chem..

[19] Kalinichenko VV (2003). Ubiquitous expression of the forkhead box M1B transgene accelerates proliferation of distinct pulmonary cell types following lung injury. J. Biol. Chem..

[20] Tan Y, Yoshida Y, Hughes DE, Costa RH (2006). Increased expression of hepatocyte nuclear factor 6 stimulates hepatocyte proliferation during mouse liver regeneration. Gastroenterology.

[21] Laoukili J (2005). FoxM1 is required for execution of the mitotic programme and chromosome stability. Nat. Cell Biol..

[22] Whitfield ML, George LK, Grant GD, Perou CM (2006). Common markers of proliferation. Nat. Rev. Cancer.

[23] Wang X, Kiyokawa H, Dennewitz MB, Costa RH (2002). The Forkhead Box m1b transcription factor is essential for hepatocyte DNA replication and mitosis during mouse liver regeneration. Proc. Natl. Acad. Sci. USA.

[24] Cabrera O (2006). The unique cytoarchitecture of human pancreatic islets has implications for islet cell function. Proc. Natl. Acad. Sci. USA.

[25] Miller RE (1981). Pancreatic neuroendocrinology: peripheral neural mechanisms in the regulation of the Islets of Langerhans. Endocr. Rev..

[26] Susaki EA (2014). Whole-brain imaging with single-cell resolution using chemical cocktails and computational analysis. Cell.

[27] Ahren B (2000). Autonomic regulation of islet hormone secretion--implications for health and disease. Diabetologia.

[28] Ahren B, Taborsky GJ, Porte D (1986). Neuropeptidergic versus cholinergic and adrenergic regulation of islet hormone secretion. Diabetologia.

[29] Fridolf T, Sundler F, Ahren B (1992). Pituitary adenylate cyclase-activating polypeptide (PACAP): occurrence in rodent pancreas and effects on insulin and glucagon secretion in the mouse. Cell. Tissue Res..

[30] Gilon P, Henquin JC (2001). Mechanisms and physiological significance of the cholinergic control of pancreatic beta-cell function. Endocr. Rev..

[31] Zawalich WS, Zawalich KC, Kelley GG (1995). Regulation of insulin release by phospholipase C activation in mouse islets: differential effects of glucose and neurohumoral stimulation. Endocrinology.

[32] Filipsson K, Sundler F, Hannibal J, Ahren B (1998). PACAP and PACAP receptors in insulin producing tissues: localization and effects. Regul. Pept..

[33] Karlsson S, Sundler F, Ahren B (1998). Insulin secretion by gastrin-releasing peptide in mice: ganglionic versus direct islet effect. Am. J. Physiol..

[34] Zhang H (2006). The FoxM1 transcription factor is required to maintain pancreatic beta-cell mass. Mol. Endocrinol..

[35] Ackermann Misfeldt A, Costa RH, Gannon M (2008). Beta-cell proliferation, but not neogenesis, following 60% partial pancreatectomy is impaired in the absence of FoxM1. Diabetes.

[36] Zhang H (2010). Gestational diabetes mellitus resulting from impaired beta-cell compensation in the absence of FoxM1, a novel downstream effector of placental lactogen. Diabetes.

[37] Davis DB (2010). FoxM1 is up-regulated by obesity and stimulates beta-cell proliferation. Mol. Endocrinol..

[38] Kawano Y (2016). Colonic Pro-inflammatory macrophages cause insulin resistance in an intestinal Ccl2/Ccr2-dependent manner. Cell Metab..

[39] Richins CA (1945). The innervation of the pancreas. J. Comp. Neurol..

[40] Duttaroy A (2004). Muscarinic stimulation of pancreatic insulin and glucagon release is abolished in m3 muscarinic acetylcholine receptor-deficient mice. Diabetes.

[41] Jain S (2013). Chronic activation of a designer G(q)-coupled receptor improves beta cell function. J. Clin. Invest..

[42] Gregersen S, Ahren B (1996). Studies on the mechanisms by which gastrin releasing peptide potentiates glucose-induced insulin secretion from mouse islets. Pancreas.

[43] Roesler R, Kent P, Luft T, Schwartsmann G, Merali Z (2014). Gastrin-releasing peptide receptor signaling in the integration of stress and memory. Neurobiol. Learn. Mem..

[44] Wahl MA, Landsbeck EA, Ammon HP, Verspohl EJ (1992). Gastrin-releasing peptide: binding and functional studies in mouse pancreatic islets. Pancreas.

[45] Ahren B (2009). Islet G protein-coupled receptors as potential targets for treatment of type 2 diabetes. Nat. Rev. Drug Discov..

[46] Vaudry D (2000). Pituitary adenylate cyclase-activating polypeptide and its receptors: from structure to functions. Pharmacol. Rev..

[47] Winzell MS, Ahren B (2007). Role of VIP and PACAP in islet function. Peptides.

[48] Xu G, Stoffers DA, Habener JF, Bonner-Weir S (1999). Exendin-4 stimulates both beta-cell replication and neogenesis, resulting in increased beta-cell mass and improved glucose tolerance in diabetic rats. Diabetes.

[49] Kulkarni RN, Mizrachi EB, Ocana AG, Stewart AF (2012). Human beta-cell proliferation and intracellular signaling: driving in the dark without a road map. Diabetes.

[50] Tiwari, S. et al. Definition of a Skp2-c-Myc pathway to expand human beta-cells. *Sci. Rep*. **6**, 28461 (2016). 10.1038/srep28461 PMC4933882 27380896

[51] Singer M (1952). The influence of the nerve in regeneration of the amphibian extremity. Q. Rev. Biol..

[52] Lebowitz P, Singer M (1970). Neurotrophic control of protein synthesis in the regenerating limb of the newt, Triturus. Nature.

[53] Kato H, Shimazu T (1983). Effect of autonomic denervation on DNA synthesis during liver regeneration after partial hepatectomy. Eur. J. Biochem..

[54] Hara M (2003). Transgenic mice with green fluorescent protein-labeled pancreatic beta -cells. Am. J. Physiol. Endocrinol. Metab..

[55] Ishigaki Y (2005). Dissipating excess energy stored in the liver is a potential treatment strategy for diabetes associated with obesity. Diabetes.

[56] Suzuki T (2011). Interleukin-6 enhances glucose-stimulated insulin secretion from pancreatic beta-cells: potential involvement of the PLC-IP3-dependent pathway. Diabetes.

[57] Matsumoto S (2006). Effects of synthetic antifreeze glycoprotein analogue on islet cell survival and function during cryopreservation. Cryobiology.

[58] Imai J (2006). Cold exposure suppresses serum adiponectin levels through sympathetic nerve activation in mice. Obesity.

[59] Takahashi R (2007). Efficient and controlled gene expression in mouse pancreatic islets by arterial delivery of tetracycline-inducible adenoviral vectors. J. Mol. Endocrinol..

[60] Bussolati G (1982). A celloidin bag for the histological preparation of cytologic material. J. Clin. Pathol..

