# Supplementary material for: Neuronal signals regulate obesity induced β-cell proliferation by FoxM1 dependent mechanism
Source: Nat Commun. 2017 Dec 5;8:1930. doi: 10.1038/s41467-017-01869-7 (PMC5717276; doi:10.1038/s41467-017-01869-7)
Supplement: Supplementary file 1 — Supplementary Information [file 41467_2017_1869_MOESM1_ESM.pdf]

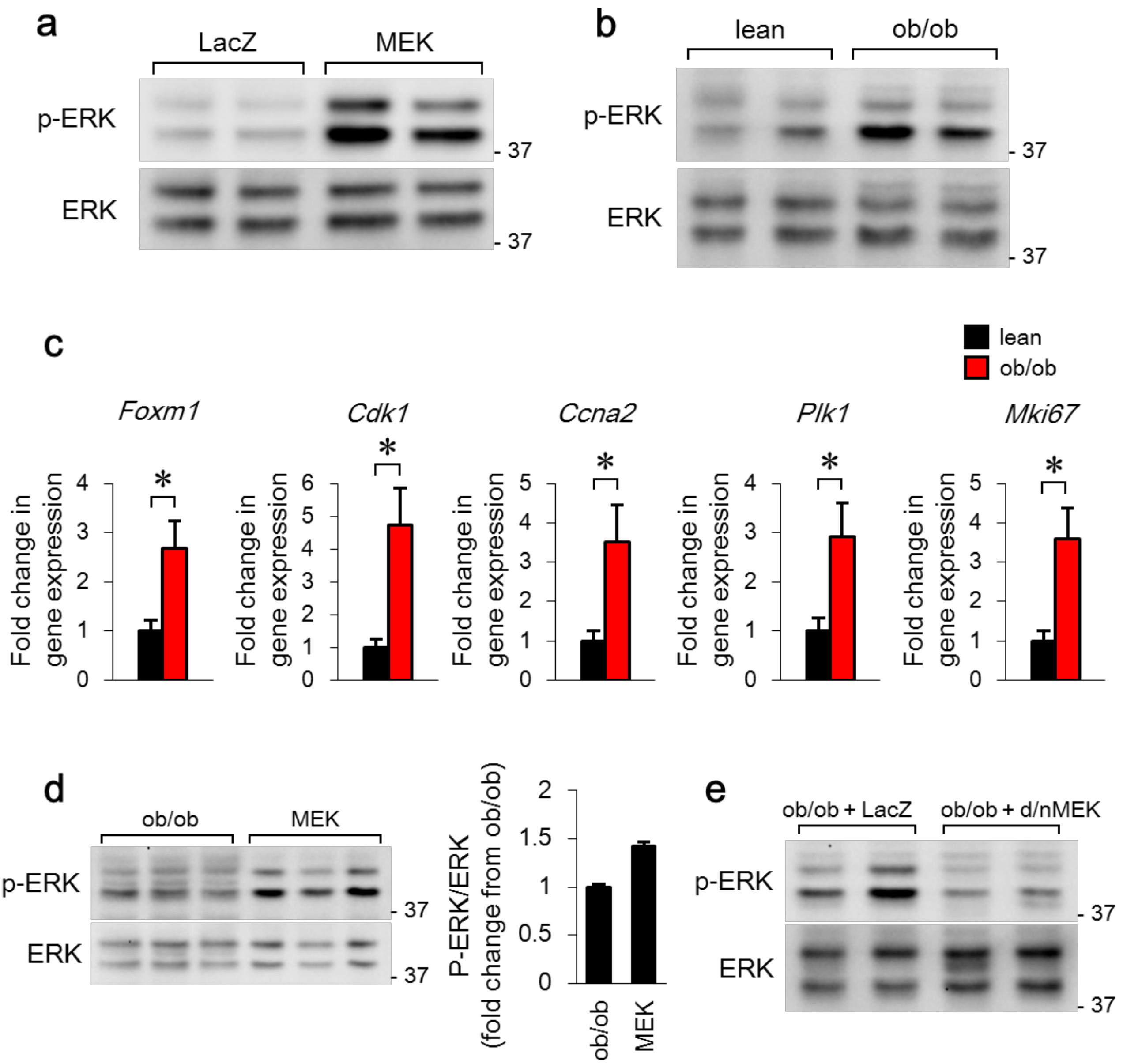

Supplementary Figure 1

Expressions of *Foxm1* and target genes as well as that of the *Mki67* gene are increased in islets along with hepatic ERK activation in ob/ob mice. (**a**) Hepatic ERK phosphorylation of L-MEK-mice or LacZ-injected mice on day 3 after adenoviral administration. (**b**) Hepatic ERK phosphorylation of 6 week-old ob/ob mice or lean littermates. (**c**) Expressions of *Foxm1* and its target genes as well as that of the *Mki67* gene in islets of 6-week-old ob/ob mice or lean littermates are shown. Data are presented as means ± s.e.m. *P < 0.05, assessed by unpaired t test. n = 4.
(**d**) Hepatic ERK phosphorylation of ob/ob mice or L-MEK-mice. The right graph shows quantitative data. n = 3. (**e**) Hepatic ERK phosphorylation of d/nMEK- or LacZ-injected ob/ob mice.

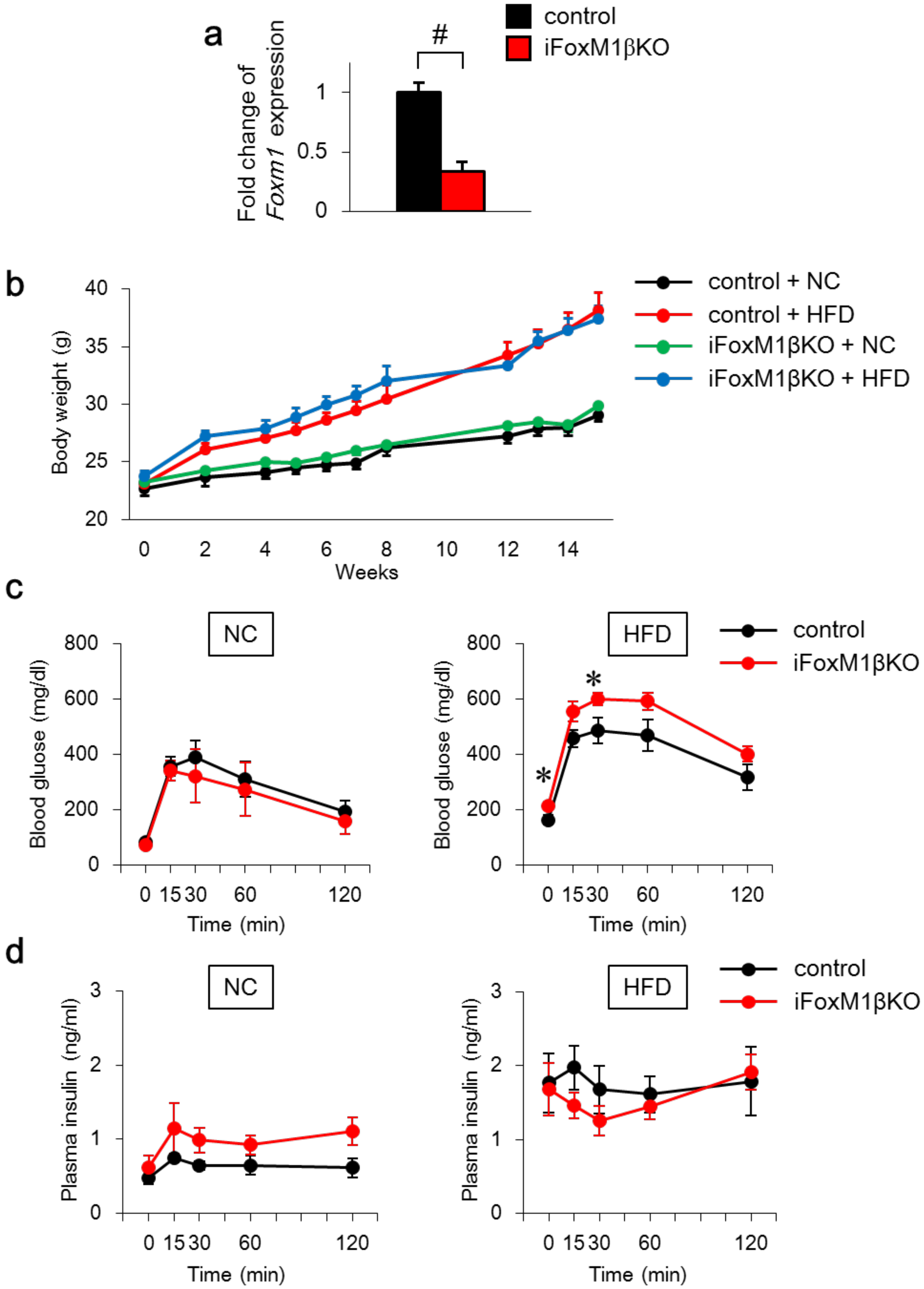

Supplementary Figure 2

The phenotypes of iFoxM1βKO mice. (**a**) Knockout deficiency of *Foxm1* in islets of iFoxM1βKO mice, on day 7 after the completion of tamoxifen injections. (**b**) Body weight changes of NC- and HFD-fed iFoxM1βKO mice or control littermates. (**c**) Blood glucose levels of FoxM1βKO mice and control littermates, after 12 weeks of HFD or NC feeding, during the glucose tolerance test. (**d**) Plasma insulin levels of FoxM1βKO mice and control littermates, after 12 weeks of HFD or NC feeding, during the glucose tolerance test. Data are presented as means ± s.e.m. *P < 0.05, #P < 0.01, assessed by unpaired t test. n = 4 (**a**), n = 4 to 10 (**b**), n = 3 to 6 (**c, d**).

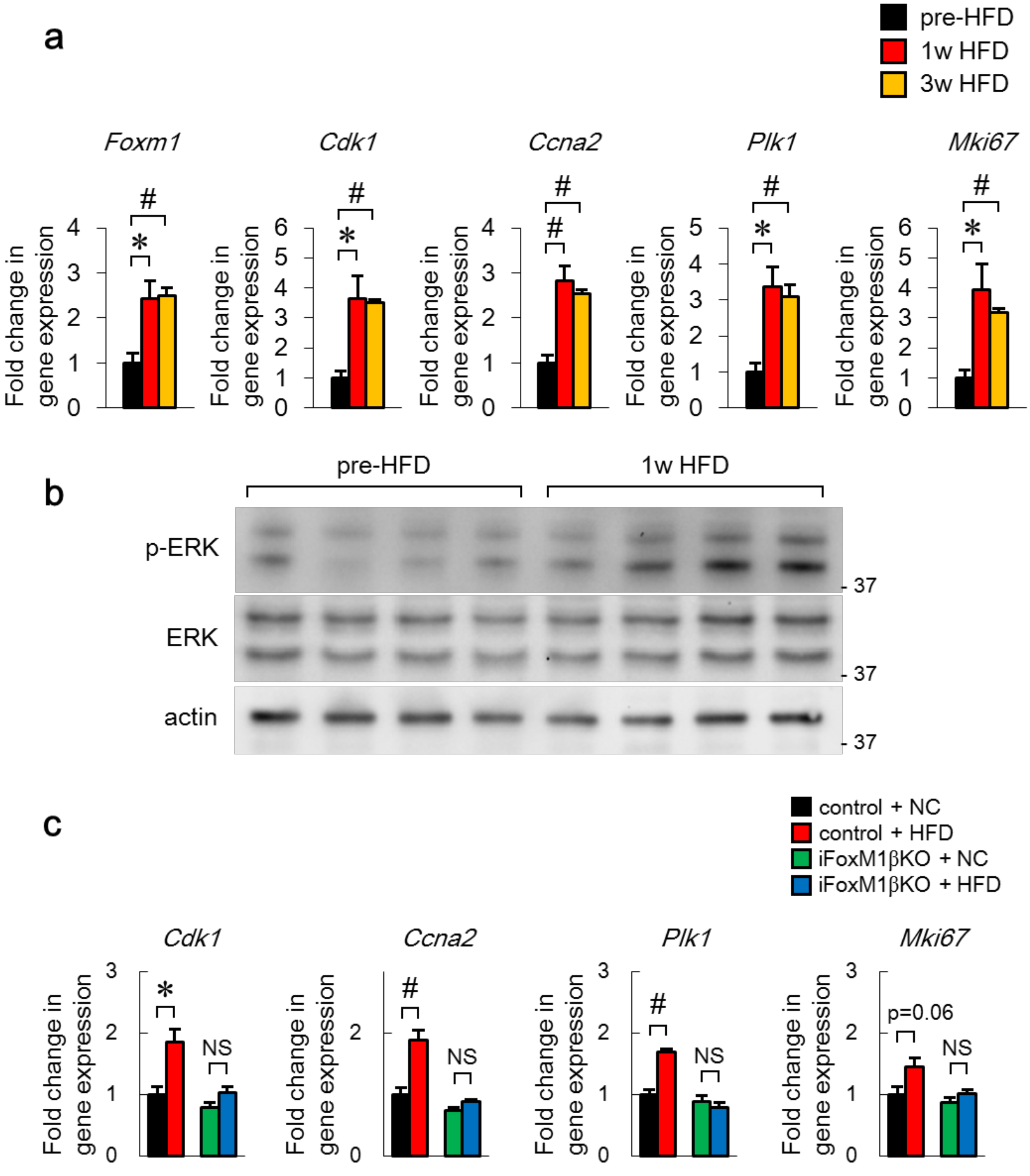

Supplementary Figure 3

Up-regulations of FoxM1 target genes and the *Mki67* gene in islets induced by one-week HFD feeding are blocked in iFoxM1βKO mice. (**a**) Expressions of *Foxm1* and its target genes as well as that of the *Mki67* gene in islets of C57BL/6N mice after HFD feeding. (**b**) Hepatic ERK phosphorylation of C57BL/6N mice after one week of HFD feeding and before HFD feeding (pre-HFD). (**c**) Expressions of FoxM1 target genes and that of the *Mki67* gene in islets of iFoxM1βKO or control littermates, after one week of HFD or NC feeding. Data are presented as means ± s.e.m. *P < 0.05, #P < 0.01, assessed by unpaired t test. n = 4 or 5 (**a**), n = 3 to 6 (**c**).

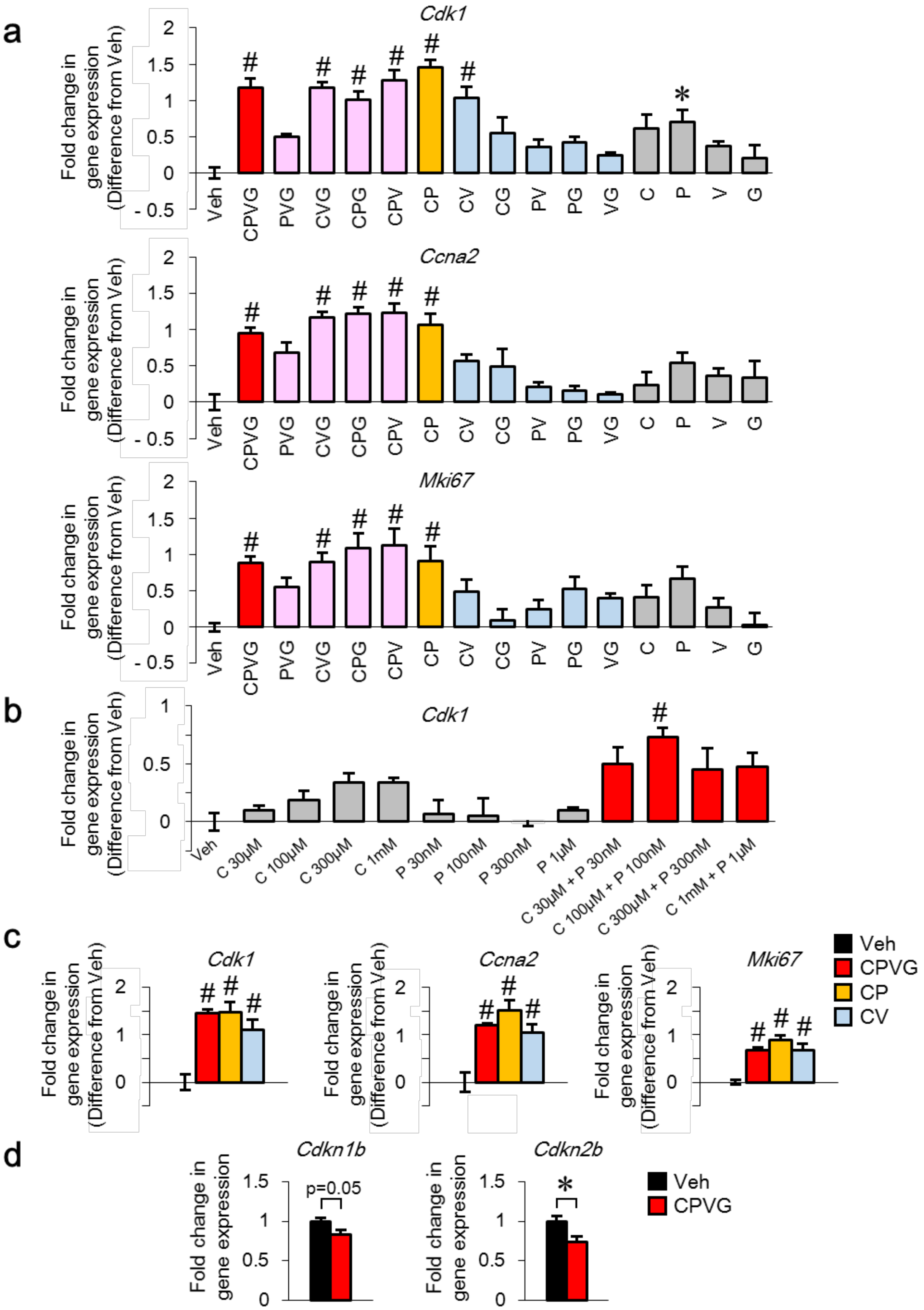

Supplementary Figure 4

Treatments combining carbachol with either PACAP or VIP increase the expressions of FoxM1 target genes and that of the *Mki67* gene in isolated islets. (**a**) Expressions of FoxM1 target genes and that of the *Mki67* gene in vagal factor-treated islets isolated from SD rats. (**b**) Expressions of *Cdk1* in islets isolated from SD rats, treated with each of the indicated concentrations of C alone, P alone, or the combination of C and P. (**c**) Expressions of FoxM1 target genes and that of the *Mki67* gene in CPVG-, CP- and CV-treated islets, isolated from C57B/6N mice. (**d**) Expressions of *Cdkn1b* and *Cdkn2b* in CPVG-treated islets isolated from C57BL/6N mice. Gene expressions of vehicle-treated islets (Veh) served as controls. Data are presented as means ± s.e.m. *P < 0.05, #P < 0.01, assessed by one-way ANOVA (**a, b**) or unpaired t test (**c, d**). n = 5 or 6 (**a**), n = 3 to 6 (**b**), n = 4 or 5 (**c**), n = 5 (**d**).

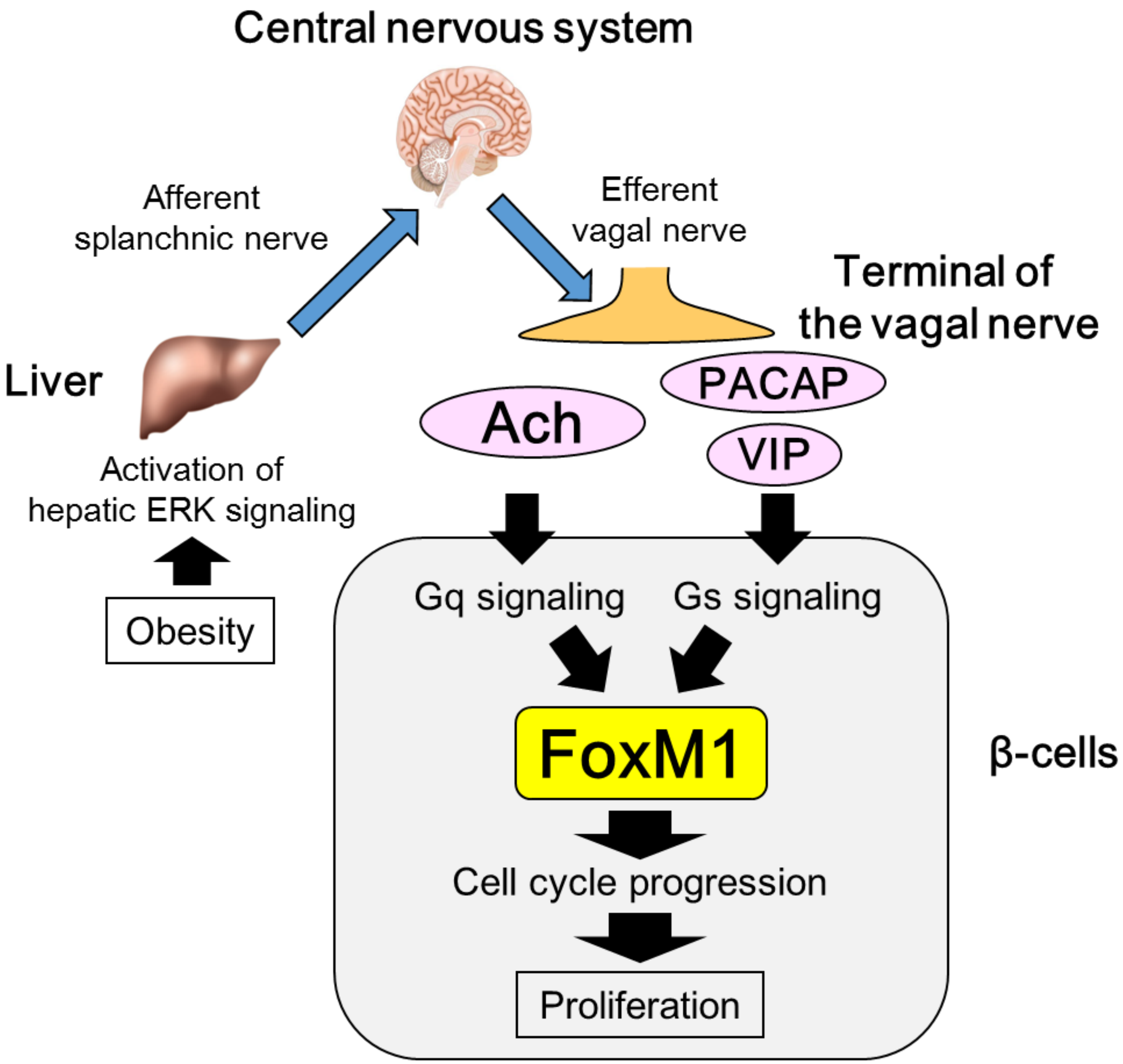

Supplementary Figure 5
Proposed molecular mechanism of β-cell proliferation induced by FoxM1 activation, mediated by vagal factors via the liver-pancreas neuronal relay, during obesity development.

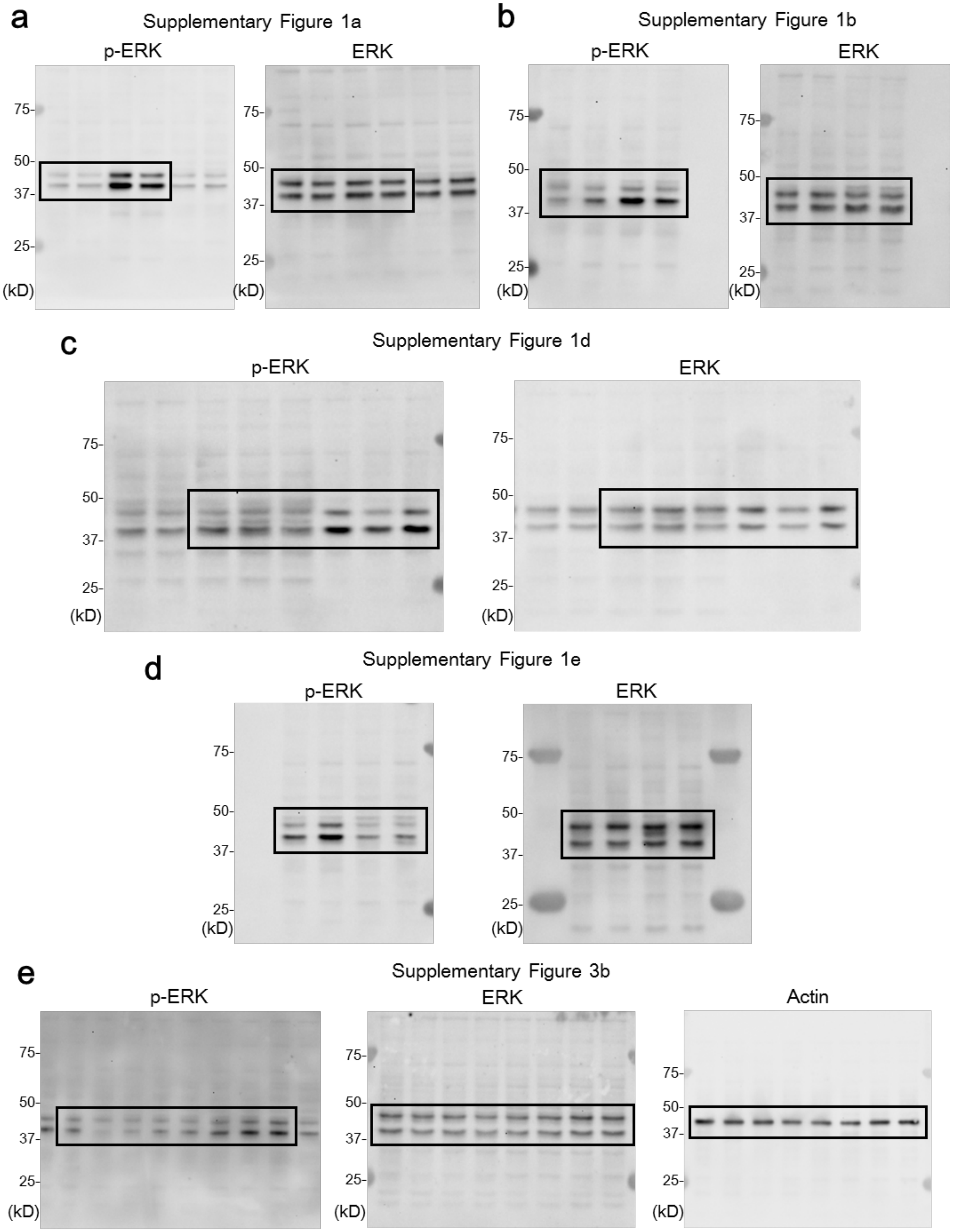

Supplementary Figure 6

Uncropped scans of the immunoblots for Supplemental Figure 1a, 1b, 1d, 1e, and 3b.

Name of the pathway	Number of genes	p-value	Zscore
Mm_Complement_and_Coagulation_Cascades_KEGG	49	0	9.656881004
Mm_Inflammatory_Response_Pathway	26	3.30E-12	6.964324704
Mm_DNA_replication_Reactome	50	1.98E-09	5.999469842
Mm_Ribosomal_Proteins	126	2.79E-08	5.554150507
Mm_Cell_Cycle_KEGG	106	2.80E-07	5.13623703
Mm_Complement_Activation_Classical	12	6.25E-07	4.983333397
Mm_Nucleotide_GPCRs	14	1.03E-05	4.411262296
Mm_Striated_muscle_contraction	30	1.44E-05	4.337583038
Mm_Prostaglandin_synthesis_regulation	23	9.56E-05	3.901485993
Mm_Blood_Clotting_Cascade	13	1.11E-04	3.865389276
Mm_IL-3_NetPath_15	167	2.66E-04	3.646496808
Mm_Focal_adhesion_KEGG	258	4.21E-04	3.526311043
Mm_Matrix_Metalloproteinases	16	6.28E-04	3.419072674
Mm_Electron_Transport_Chain	97	8.13E-04	3.348246787
Mm_S1P_Signaling	37	1.26E-03	3.225938246
Mm_Nucleotide_Metabolism	24	2.34E-03	3.043743392
Mm_IL-9_NetPath_20	38	7.23E-03	2.685902487
Mm_Eicosanoid_Synthesis	17	8.82E-03	2.618765008
Mm_Apoptosis	117	1.01E-02	2.57272684
Mm_Triacylglyceride_Synthesis_BiGCaT	38	1.52E-02	2.427472351
Mm_GPCRDB_Class_A_Rhodopsin-like	79	2.06E-02	2.314960402
Mm_B_Cell_Receptor_NetPath_12	230	2.11E-02	2.306240141
Mm_GPCRDB_Other	51	0.02137	2.301458286
Mm_Proteasome_Degradation	121	2.24E-02	2.282873819
Mm_IL-4_NetPath_16	100	2.44E-02	2.250262541
Mm_Cell_Cycle-G1_to_S_control_Reactome	93	2.76E-02	2.202745025
Mm_Smooth_muscle_contraction	225	0.057745	1.897631754
Mm_Osteoclasts	14	6.86E-02	1.820805622
Mm_T-Cell-Receptor_NetPath_11	179	7.08E-02	1.80670769
Mm_Heme_Biosynthesis	11	0.082226	1.737914218
Mm_Integrin-mediated_cell_adhesion_KEGG	148	0.11118	1.592910329
Mm_Glycolysis_and_Gluconeogenesis	54	0.134558	1.496367613
Mm_Adipogenesis	172	0.135752	1.491796743
Mm_Gluthation_Metabolism_KEGG	21	0.143973	1.461154334
Mm_IL-7_NetPath_19	72	0.182609	1.332764218
Mm_Delta-Notch_NetPath_3	120	0.183107	1.331248014
Mm_IL-6_NetPath_18	148	0.194489	1.297414668
Mm_IL-2_NetPath_14	131	0.209363	1.255318137
Mm_Regulation_of_Actin_Cytoskeleton_KEGG	218	0.232539	1.19384523
Mm_One-carbon_metabolism	40	0.240003	1.174978832
Mm_Mitochondrial_fatty_acid_betaoxidation	20	0.261327	1.123260044
Mm_Biogenic_Amine_Synthesis	13	0.276539	1.088126299
Mm_IL-5_NetPath_17	103	0.315277	1.004209942
Mm_Androgen-Receptor_NetPath_2	178	0.345283	0.943778126
Mm_Fatty_Acid_Beta_Oxidation_1_BiGCaT	40	0.356741	0.921591967
Mm_GPCRDB_Class_B_Secretin-like	12	0.375717	0.885814606
Mm_Peptide_GPCRs	25	0.430497	0.788340805
Mm_TNF-alpha-NF-kB_NetPath_9	288	0.45166	0.752650816

Name of the pathway	Number of genes	p-value	Zscore
Mm_TGF_Beta_Signaling_Pathway	63	0.475206	0.714034194
Mm_Oxidative_Stress	34	0.48863	0.692489719
Mm_Translation_Factors	95	0.510544	0.657990517
Mm_Statin_Pathway_PharmGKB	21	0.527837	0.631310582
Mm_Selenium-metabolism_Selenoproteins	66	0.530558	0.627155055
Mm_Insulin_Signaling	237	0.555214	0.589964472
Mm_Fatty_Acid_Beta_Oxidation_Meta_BiGCaT	47	0.592474	0.535253978
Mm_Alanine_and_aspartate_metabolism_KEGG	14	0.616313	0.501081918
Mm_IL-1_NetPath_13	60	0.836666	0.206159508
Mm_Hedgehog_Netpath_10	28	0.886807	0.142345136
Mm_Alpha6-Beta4-Integrin_NetPath_1	97	0.917448	0.103648375
Mm_EGFR1_NetPath_4	272	0.920464	9.98E-02
Mm_Fas_Pathway_and_Stress_Induction_of_HSP_Regulation_Biocarta	57	0.984587	0.01931911
Mm_Calcium_regulation_in_cardiac_cells	197	0.885494	-0.14400846
Mm_Pentose_Phosphate_Pathway	10	0.874124	-0.15842222
Mm_MAPK_signaling_pathway_KEGG	215	0.860705	-0.17547663
Mm_G13_Signaling_Pathway	59	0.804017	-0.24815122
Mm_Kit-Receptor_NetPath_6	106	0.686718	-0.40331356
Mm_Fatty_Acid_Synthesis_BiGCaT	30	0.649672	-0.45421828
Mm_Wnt_NetPath_8	154	0.600842	-0.52318985
Mm_Cholesterol_Biosynthesis	31	0.597987	-0.52729767
Mm_Krebs-TCA_Cycle	46	0.59375	-0.53340927
Mm_Signaling_of_Hepatocyte_Growth_Factor_Receptor_Biocarta	58	0.589209	-0.53998291
Mm_MAPK_Cascade	53	0.587082	-0.54306884
Mm_ESC_Pluripotency_Pathways	158	0.53804	-0.61578004
Mm_Nuclear_Receptors	39	0.519718	-0.64378063
Mm_Notch_signaling_KEGG	29	0.491376	-0.68812241
Mm_Nuclear_receptors_in_lipid_metabolism_and_toxicity	21	0.474887	-0.71454955
Mm_Glycogen_Metabolism	33	0.455388	-0.74646205
Mm_p38_MAPK_signaling_pathway	48	0.44445	-0.76470101
Mm_G_Protein_Signaling	138	0.432529	-0.78487123
Mm_Tryptophane_Metabolism_KEGG	41	0.431311	-0.78695102
Mm_Fatty_Acid_Beta_Oxidation_3_BiGCaT	10	0.423721	-0.79998201
Mm_Urea_cycle_and_metabolism_of_amino_groups_KEGG	22	0.387627	-0.86392917
Mm_RNA_transcription_Reactome	62	0.36714	-0.90184441
Mm_mRNA_processing_binding_Reactome	745	0.351944	-0.930826
Mm_HSP70_and_Apoptosis	30	0.232434	-1.19411191
Mm_Ovarian_Infertility_Genes	38	0.186494	-1.32102328
Mm_Small_ligand_GPCRs	10	9.44E-02	-1.67284733
Mm_Wnt_Signaling	98	0.085966	-1.71707461
Mm_Hypertrophy_model	20	6.90E-02	-1.81861192
Mm_Acetylcholine_Synthesis	12	3.88E-02	-2.06649866
Mm_TGF-beta-Receptor_NetPath_7	227	3.05E-02	-2.16379703
Mm_Id_NetPath_5	66	6.26E-03	-2.73386594
Mm_Circadian_Exercise	85	1.12E-03	-3.25798697

Supplementary Table 1

Results of pathway analysis of microarray data obtained from the islets of L-MEK-mice compared to that obtained from the islets of LacZ-injected mice.

	forward (5' to 3')	reverse (5' to 3')
Mouse Foxm1	GCTCCATAGAAATGTGACCATC	AACCTTCACTGAGGGCTGTAAC
Mouse Cdk1	AAGAACCTGGACGAGAACG	GTCATCAAAGTACGGGTGCT
Mouse Ccna2	CCTTAAGTACCTGCCTTCACTC	ACAAGGCTTAAGACTCTCCA
Mouse Plk1	ACGGCACCGTGCAGATTA	AGGCGGTACGTTTGGAAGTC
Mouse Mki67	AGTCTCTGGAGAGTCTGATGTTA	ACTTCTTGGTGCATACAATGTC
Mouse Cdkn1b	CGACGCCAGACGTAAACAG	CAGTGCTTATACAGGATGTCCA
Mouse Cdkn2b	CGCTGCCACTGGAGATTGA	CGTGCTTGCAGTCTTCCTAGATG
Mouse Actb	GATGCCCTGAGGCTCTT	TGTGTTGGCATAGAGGTCTTTAC
Rat Cdk1	CACGTCAAGAACCTGGATG	GATCTGATTGTCCAAGTCGTC
Rat Ccna2	GCTCTCTACACAGTCACAGGACA	GGTGAAGGTCCATGAGACAAG
Rat Mki67	ATCCAGATGTTAGGCGTTTGAG	ACAGTTTCACTTTTCTGGTGACTTC
Rat Actb	CCTAGCACCATGAAGATCAAGA	GTGTAAAACGCAGCTCAGTAAC

Supplementary Table 2
Sequences of quantitative RT-PCR primers.
